# Enhanced generation of influenza-specific tissue resident memory CD8 T cells in NK-depleted mice

**DOI:** 10.1038/s41598-021-88268-7

**Published:** 2021-04-26

**Authors:** David L. Rose, Katie L. Reagin, Kimberly E. Oliva, S. Mark Tompkins, Kimberly D. Klonowski

**Affiliations:** 1 Department of Shared Resources, Beckman Research Institute of the City of Hope, Duarte, CA 91010 USA; 2 Department of Biology, University of North Carolina at Charlotte, Charlotte, NC 28223 USA; 3 Department of Cellular Biology, University of Georgia, Athens, GA 30602 USA; 4 Department of Infectious Diseases, University of Georgia, Athens, GA 30602 USA; 5 Center for Vaccines and Immunology, University of Georgia, Athens, GA 30602 USA

**Keywords:** Immunological memory, Immunology

## Abstract

Natural Killer (NK) cells are among the first effectors to directly contact influenza and influenza-infected cells and their activation affects not only their intrinsic functions, but also subsequent CD8^+^ T cell responses. We utilized a NK cell depletion model to interrogate the contribution of NK cells to the development of anti-influenza CD8^+^ T cell memory. NK cell ablation increased the number of influenza-specific memory CD8^+^ T cells in the respiratory tract and lung-draining lymph node. Interestingly, animals depleted of NK cells during primary influenza infection were protected as well as their NK-intact counterparts despite significantly fewer reactivated CD8^+^ T cells infiltrating the respiratory tract after lethal, heterosubtypic challenge. Instead, protection in NK-deficient animals seems to be conferred by rapid reactivation of an enlarged pool of lung tissue-resident (T_RM_) memory cells within two days post challenge. Further interrogation of how NK cell ablation enhances respiratory T_RM_ indicated that T_RM_ development is independent of global and NK cell derived IFN-γ. These data suggest that reduction in NK cell activation after vaccination with live, non-lethal influenza virus increases compartmentalized, broadly protective memory CD8^+^ T cell generation and decreases the risk of CD8^+^ T cell-mediated pathology following subsequent influenza infections.

## Introduction

Seasonal influenza A viruses cause significant morbidity worldwide, resulting in high rates of influenza-associated hospitalization^1,2^. Current influenza vaccines target humoral responses to strain-specific hemagglutinin (HA) but weakly elicit cross-protective immunity against divergent and mutated viruses^3^. However, anti-influenza CD8^+^ T cells recognize conserved, internal viral proteins and clear the virus infection. Therefore, targeting the generation of anti-viral CD8^+^ memory T cells (T_mem_) may provide a better alternative to combat evolving influenza strains^4^. The presence of tissue resident memory cells (T_RM_) within the respiratory tract has been correlated with increased protection against heterologous influenza infection^5^. However, respiratory T_RM_ wane with time, coordinate with protection against secondary influenza infection^5^. Thus, further understanding the factors which contribute to the generation of long-lived T_RM_ in the respiratory tract may provide an exploitative mechanism to enhance protection against seasonal influenza infections.

While the signals required for the generation of T_mem_ cells in the respiratory tract are not fully understood, CD8^+^ T cell fate determination is linked strength of TCR signaling, duration of T cell-APC interactions, co-stimulation, and CD4 help^6^. Moreover, integration of these signals and cytokines in the priming environment tune PI3K and mTOR signaling pathways towards either an effector or T_mem_ cell differentiation program^6^. Additionally, circumstances surrounding the activation of an individual T cell can be unique, with different clones of a single specificity and progeny of a single naïve T cell capable of generating effector and diverse T_mem_ lineages^7^. All of the factors driving the T_mem_ differentiation process are contextual, with the pathogen and specific priming microenvironment dictating T_mem_ potential. Importantly, strong inflammation promotes terminal differentiation at the expense of memory formation in part via the cytokines IL-12 and IFN-γ^8–10^ via differential regulation the transcription factors T-bet^11^ and Eomesodermin^12^. Knowledge of how immune cells supporting the inflammatory response contribute to the CD8^+^ T cell priming environment and resultant T_mem_ development could be used to improve influenza vaccine design.

NK cells precede CD8^+^ T cells in the respiratory tract after influenza infection and can influence anti-viral adaptive immunity^13^. Indeed, NK cells can directly suppress the proliferation of recently activated CD8^+^ T cells^14,15^, eliminate CD8^+^ T cells either infected or not protected by the anti-viral state^13,16–18^ and affect the overall magnitude of CD8^+^ T cell priming through direct elimination of virus^19^. Moreover, NK cells can recognize and lyse activated CD4^+^ helper T cells, subsequently limiting the CD8^+^ T cell response to viral infection^20^. It is therefore likely that NK cells could directly or indirectly influence the formation of CD8^+^ T_mem_, making them an important variable to consider for elicitation of cross-protective CD8^+^ T cell immunity.

Here, we demonstrate that systemic depletion of NK cells during primary influenza A infection results in a numerically larger pool of influenza-specific CD8^+^ T_mem_ in the lung and lung-draining lymph nodes. Importantly, the former pool is enriched for T_RM_ positioned within the lung parenchyma and outside of the associated vasculature. NK-depleted and intact animals were equally protected from a heterosubtypic viral challenge; however, significantly fewer peripherally reactivated CD8^+^ T_mem_ were observed in the lungs of NK-depleted animals. Instead, viral titers were likely controlled by the rapid reactivation of an enriched T_RM_ population pre-positioned in the lungs. Upon ex vivo peptide stimulation memory CD8^+^ T Cells from both NK-sufficient and deficient animals produced similar amounts of anti-viral cytokines, including IFN-γ, on a per cell basis, however the enlarged T_RM_ pool in the NK-deficient mice allows for greater overall production of anti-viral cytokines early after viral challenge. Elimination of global IFN-γ did not enhance T_RM_ formation, suggesting NK cell-derived IFN-γ does not mediate this effect. In summary, our studies suggest that tuning NK cell activation and function may present a mechanism to elicit optimal CD8^+^ T_RM_ during vaccination.

## Results

### Systemic depletion of NK cells results in numerically increased CD8^+^ T_mem_

Innate immune cells are first responders, decreasing antigen load and producing cytokines, which can modulate subsequent adaptive immune response^19,21^. Indeed, NK cells have been shown to impact the magnitude and longevity of subsequent anti-viral T cell responses^19,20,22,23^. However, it is unclear how NK cells modulate anti-influenza CD8^+^ T cell responses, specifically their differentiation into memory subsets and resultant recall responses in vivo. We administered the monoclonal antibody PK136 (anti-NK1.1), an isotype control (IgG), or PBS control to deplete NK cells 1 day prior to, and ending 3 days post, sub-lethal infection with the low pathogenicity influenza A virus x31. This protocol depletes NK cells in the blood, lung, and secondary lymphoid organs > 90% through 5 days post infection (dpi) (Supplementary Fig. S2 and data not shown), the time over which NK cells accumulate and become activated within the lung (Supplementary Fig. S1), with no effect on viral load (Supplementary Fig. S2). While we observe restoration of NK cells in the blood and secondary lymphoid organs by 35dpi (Supplementary Fig. S2 and data not shown), NK cells numbers are still significantly reduced in the lung as far out as 35dpi, suggesting this depletion protocol results in a protracted depletion of lung NK cells. While the anti-NK1.1 mAb can deplete both NK1.1^+^ NKT cells^24^ and NK1.1^+^ ILC-1s^25^, we determined that NKT cells were neither significantly expanded nor activated in our model of influenza infection (Supplementary Fig. S1).

**Figure 1:**
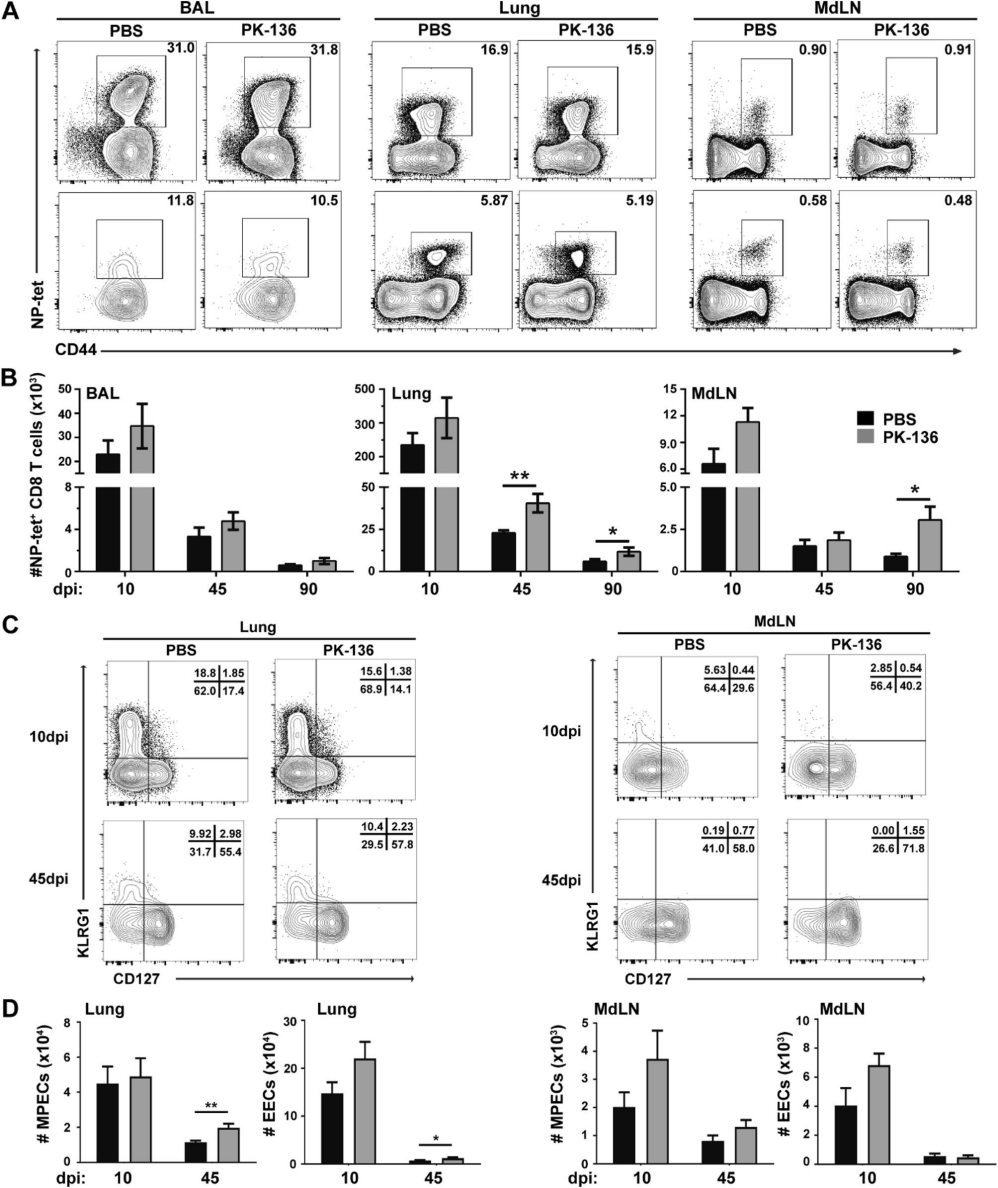
Anti-influenza CD8^+^ T cells are numerically increased following NK cell depletion. C57BL/6 mice were infected with 10^3^ PFU x31 and administered PBS or the NK depleting antibody at −1, 1, and 3 dpi. (**A**) Representative flow of activated (CD44^hi^), NP-tet^+^ cells recovered from the BAL, lung, and MdLN of animals after gating on single cells, lymphocytes and CD8^+^ cells. (**B**) The kinetics of the CD8^+^ T cell response based on the total number ± SEM of activated (CD44^hi^), NP-tet^+^ CD8^+^ T cells recovered from the BAL, lung, and MdLN of animals that were treated with PBS (black) or depleting antibody (grey) is depicted. (**C**) Representative flow of CD127 and KLRG1 expression by NP-tet^+^CD44^+^CD8^+^ T cells from the lung and MdLN of NK sufficient or depleted mice 10 and 45dpi. (**D**) The total number of CD127^+^KLRG1^-^ (MPECs) and CD127^-^KLRG1^-^ (EECs) cells in the lung and MdLN of animals treated with PBS (black) or PK136 (gray), previously gated on CD8^+^CD44^hi^NP-tet^+^ cells. Graphs represent pooled data from 3 or more independent experiments using n ≥ 3 mice/group/time point (total of 9 mice per group/time point). *p < 0.05; **p < 0.01; unpaired Student’s t-test with Holm–Sidak multiple comparisons correction.

To determine whether NK depletion affects the magnitude of the anti-influenza CD8^+^ T cell response, we monitored the accumulation of the immunodominant anti-influenza nucleoprotein (NP)-specific CD8^+^ T cells in the lung, lung airways (via bronchial alveolar lavage (BAL)), and lung-draining mediastinal lymph nodes (MdLN) after infection in the presence or absence of NK cells. Depletion of NK cells increased the numbers of NP-specific CD8^+^ T cells recovered from all respiratory-associated tissues at 10 dpi (Fig. 1A,B), the peak of the proliferative CD8^+^ T cell response, albeit not to a statistically significant level. However, this disparity increased over time in the lung where more than double the number of NP-specific CD8^+^ T cells were isolated from the respiratory tract of NK-depleted mice 45 dpi. In the MdLN we observed a similar trend, albeit slightly delayed, with nearly double the number of NP-specific CD8^+^ T cells recovered from NK-depleted mice 90 dpi (Fig. 1A,B). While the number of antigen (Ag)-specific CD8^+^ T cells isolated from the BAL of NK-depleted mice was elevated over NK-sufficient mice at all times examined, the difference between the two groups was not statistically significant. Nonetheless, these data demonstrate that depletion of NK cells numerically increased the pool of anti-influenza CD8^+^ T_mem_ in both the lung parenchyma and lung-draining MdLN after influenza infection. As demonstrated previously^19^, this suggests that NK cells limit the accumulation of CD8^+^ T_mem_; however, it’s unclear whether NK cell ablation affects the development (and/or survival) and quality of the resultant CD8^+^ T_mem_.

### NK cell depletion increases the pool of CD127 expressing memory cells following influenza infection

T_mem_ cells develop from effectors which survive the contraction phase of the immune response via IL-7 signalling^26,27^. Thus, cells with memory potential are identified by increased IL-7Rα (CD127) expression and are termed memory precursor effector cells (MPECs). Conversely, effector cells lacking CD127 expression will not survive contraction, and are thus termed short-lived effector cells (SLECs) due to their limited longevity and expression of terminal differentiation marker killer cell lectin-like receptor G1 (KLRG1)^6^. Effector cells devoid of both CD127 and KLRG1 are termed early effector cells (EECs) and have demonstrated a plasticity in their ability to transition into either SLECs or MPECs depending on the inflammatory milieu^7,28^. While it has been demonstrated that influenza infection favors development of a large EEC pool^29^, it is unknown whether NK depletion alters T_mem_ development, even at this early stage.

To determine whether NK cells affect the memory potential of Ag-specific CD8^+^ T cells, we tracked the kinetics of CD127 and KLRG1 expression on influenza-specific CD8^+^ T cells in the lung and MdLN 10 and 45 dpi. The frequency and total number of KLRG1^hi^ (both CD127^hi^ (DPECs) and CD127^lo^ (SLECs)) recovered from the lung and MdLN was equivalent regardless of NK-depletion (Fig. 1C and data not shown). However, the total number of EECs (CD127^lo^KLRG1^lo^) recovered from the lungs was elevated in NK-depleted mice at both 10 and 45 dpi (Fig. 1D). Interestingly, almost 2 × as many MPECs were recovered from the lungs of NK-depleted mice compared to NK-sufficient controls 45 dpi despite no difference at 10 dpi (Fig. 1D). Within the MdLN, although not statistically significant, both MPECs and EECs were elevated 10 dpi in NK-depleted mice, and while the EECs dropped off to equivalent levels by 45dpi, MPECs remained elevated in NK-depleted mice compared to NK-sufficient controls (Fig. 1D). Since CD127^hi^ expressing CD8^+^ T cells undergo contraction at a slower rate than their CD127^lo^ counterparts^30^, CD127^hi^ NP-specific CD8^+^ T cells isolated from NK-depleted mice would have a greater chance of surviving to memory. Indeed, comparing the total number of lung MPECs between 10 and 45 dpi suggests that MPECs may have contracted at a slower rate in NK-depleted animals (Fig. 1D). Together, these data show that NK cell depletion increases the number of MdLN and lung T_mem_ generated after influenza infection which may be attributed to a reduced contraction of lung MPECs.

### NK Cell depletion increases the number of Ag-specific CD8^+^ T_RM_

Memory cells are broadly categorized as central (T_CM_) or effector (T_EM_) memory based on distinct cell surface marker expression which influences localization^31^. While T_EM_ patrol peripheral tissues, tissue resident memory cells (T_RM_)_,_ a subset of T_EM,_ have limited migratory potential. It has long been appreciated that anti-influenza CD8^+^ T cells positioned at the infection site are crucial for heterosubtypic immunity and waning of this immunity correlates with loss of respiratory CD8^+^ T cells over time^32,33^. Recent studies have indeed attributed protective immunity against secondary viral challenge to the presence of T_RM_ whereas infiltrating memory CD8^+^ T cells are associated with immunopathology^5^.

**Figure 2:**
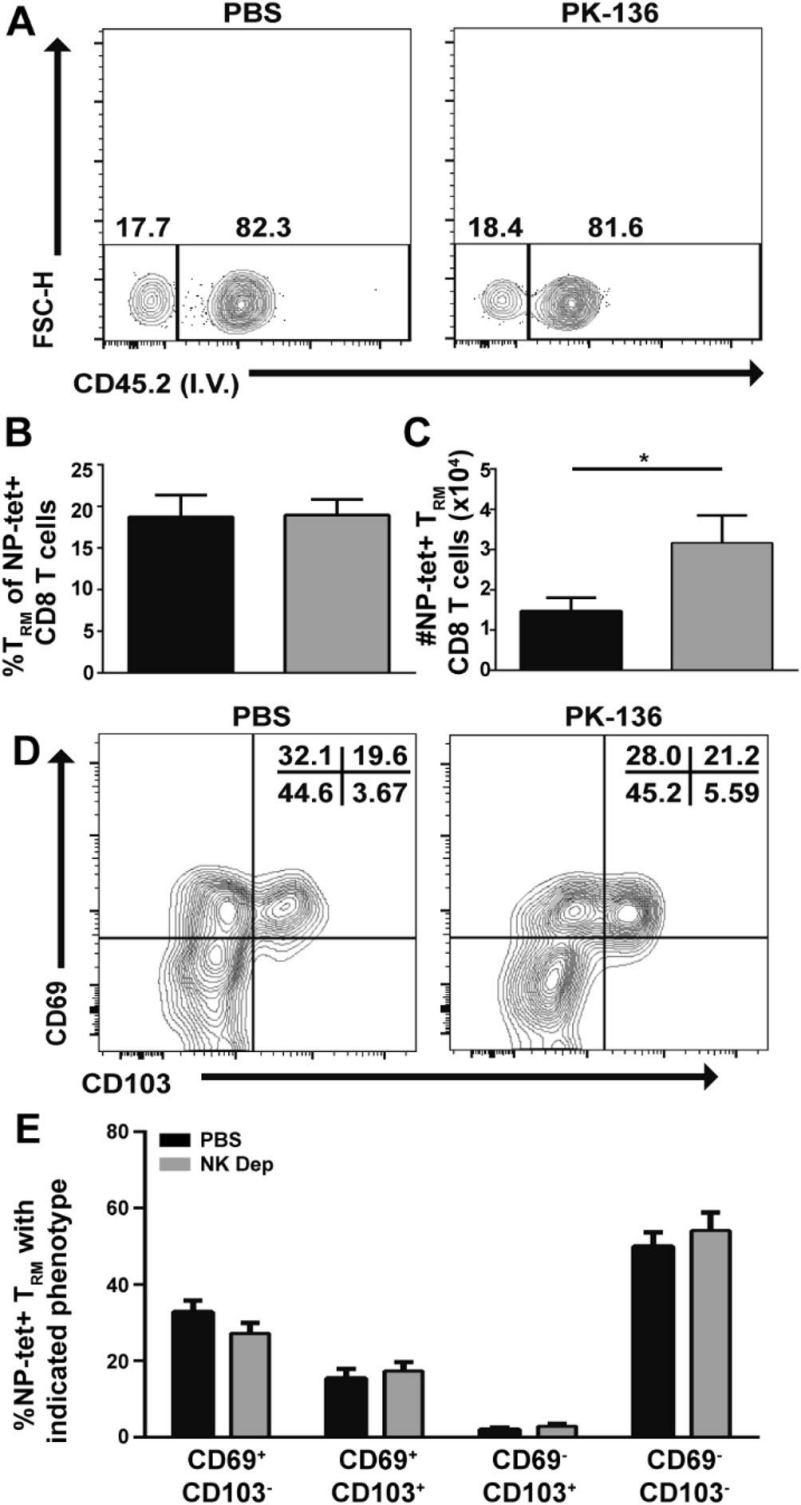
NK depletion enhances the development of lung T_RM_ following influenza infection. (A) C57BL/6 mice were infected with 10^3^ PFU x31, administered anti-NK depleting antibody at −1, 1, and 3 dpi and rested for 45 days. T_RM_ were identified as cells which did not positively stain with an anti-CD45 administered i.v. 3 min prior to euthanasia, previously gated on single cells, lymphocytes, CD8^+^CD44^+^NP-tet^+^ cells. The frequency (B), and number (C) of NP-tet^+^ T_RM_ CD8^+^ T cells ± SEM recovered from the lung of NK depleted (grey) and sufficient (black) mice are depicted. (D) Representative expression of CD103 and CD69 by lung T_RM_ cells. (E) Frequency of cells expressing CD103 or CD69 in NK-depleted (grey) or sufficient (black) mice. Graphs represent pooled data from 2 independent experiments using n ≥ 3 mice/group/time point (total of 6 mice per group/time point). *p < 0.05; unpaired Student’s t-test with Holm-Sidak multiple comparisons correction.

As NK-depleted mice have enhanced T_mem_ in the lung following influenza infection, we wanted to determine whether lung T_RM_ specifically were enhanced. Thus, we differentiated circulating from resident CD8^+^ T_mem_ in NK-depleted and sufficient mice 50 dpi using an established intravenous staining protocol^34^, which differentiates between circulating and tissue-embedded NP-specific CD8^+^ T_mem_ at a given time (Fig. 2A)^34^. We observed no difference in the frequency of CD8^+^ T_EM_ (data not shown) or T_RM_ between NK-sufficient or NK-deficient mice (Fig. 2A,B). However, due to the numerical increase in CD8^+^ T cells in the lungs of NK-depleted animals, there was an increase in the total number of T_EM_ (data not shown), and the total number of T_RM_ was coordinately increased by twofold compared to NK-sufficient animals (Fig. 2C). Additionally, we do not observe any increase in the total number of lung T_RM_ following PBS treatment or treatment with 200ug isotype control IgG (Fig S3), suggesting this enhancement in T_RM_ is in fact due to NK depletion. Despite this numerical increase in T_RM_, we observed no difference in expression of canonical T_RM_ markers CD69 or CD103 (Fig. 2D,E).

**Figure 3:**
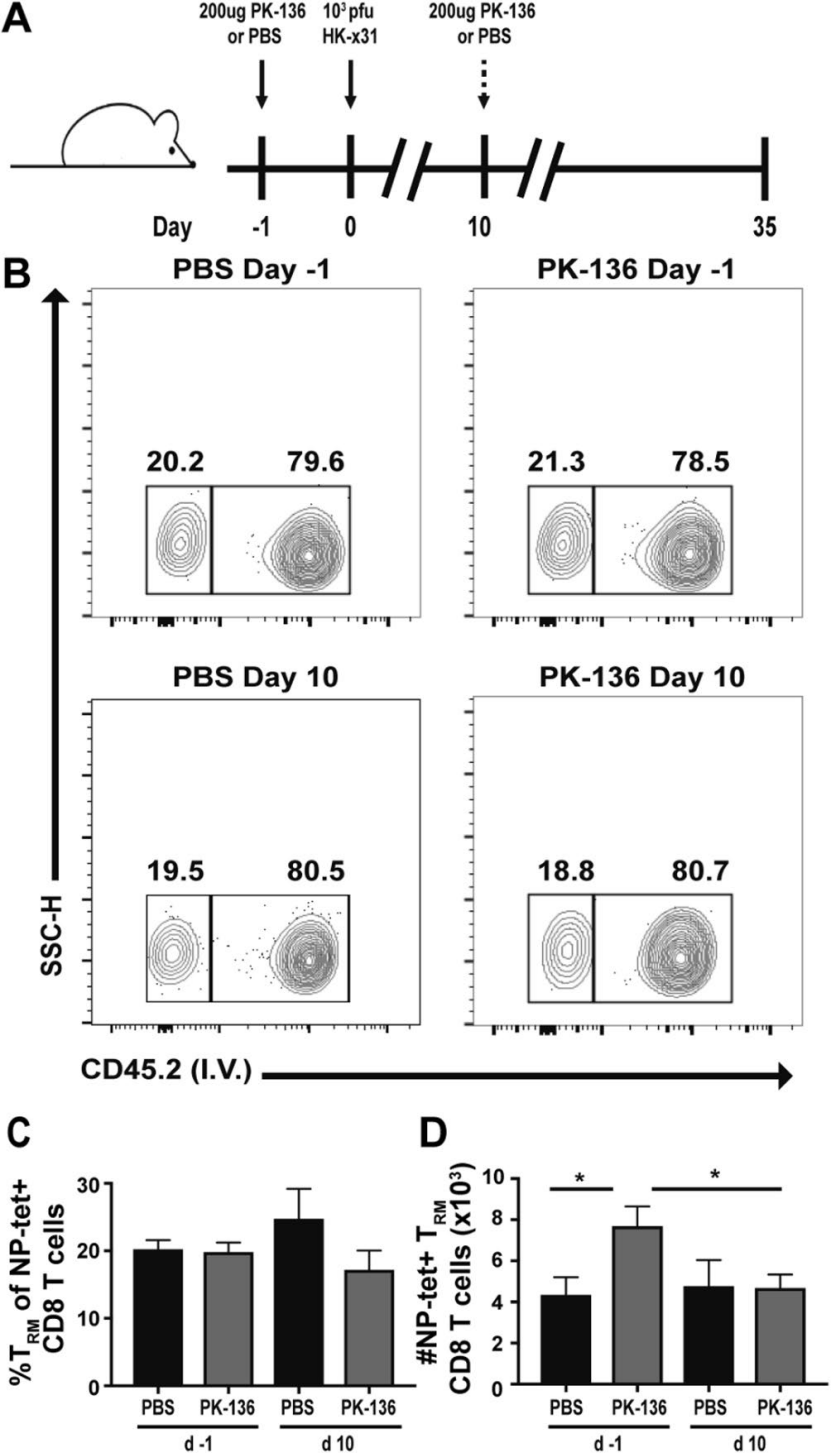
Removal of NK cells during CD8^+^ T cell priming enhances the development of lung T_RM_. (**A**) C57BL/6 mice were infected with 10^3^ PFU x31, administered anti-NK depleting antibody at −1 or 10 dpi, and sacrificed 35 dpi. T_RM_ were identified by lack of staining with an anti-CD45 antibody administered i.v. 3 min prior to euthanasia. (**B**) Representative flow of circulating (i.v.^-^) or resident (i.v.^+^) memory cells within the lung 35dpi following treatment with PBS or PK-136 on the indicated days. Previously gated on single cells, lymphocytes, CD8^+^CD44^+^NP-tet^+^ cells. The frequency (**C**) and number (**D**) of NP-tet^+^ T_RM_ CD8^+^ T cells + /-SEM recovered from the lung of NK depleted (grey; depletion protocol as indicated and described in (**A**)) vs PBS (black) are depicted. All graphs represent pooled data from 2 independent experiments with n = 5 mice/group/time point (total of 10 mice per group/time point). *p < 0.05; unpaired Student’s t-test with Holm-Sidak multiple comparisons correction.

As our NK depletion model removes circulating NK cells for approximately 35 days (Supplementary Fig. S2), NK cell alteration of T_RM_ cell numbers could be early (programmatic) or later (via enhanced survival during contraction). Therefore, we depleted NK cells at −1 or 10 dpi (Fig. 3A), where NK cells are retained during CD8^+^ T cell priming, expansion and migration and depleted only during contraction. Regardless of depletion protocol, the frequency of NP-specific CD8^+^ T_RM_ was equivalent between NK-depleted and sufficient animals (Fig. 3B,C). However, only the mice depleted of NK cells during priming, expansion and migration into tissues (at d-1) had an increase in the total number of CD8^+^ T_RM_ (Fig. 3D), indicating that the NK cells exert an early effect on CD8^+^ T cells at a time when these cells are receiving key programmatic signals, ultimately limiting the expansion of the T_RM_ pool.

### NK cell ablation during primary influenza infection leads to numerically smaller but equally protective CD8^+^ T cell recall response

Positioning of T_RM_ cells within the respiratory tract mediates protective recall responses upon secondary influenza infection^5,35^. Since NK-depletion enhances the numbers of T_mem_, and specifically T_RM_, in the respiratory tract, we wanted to determine whether this translated into a more robust recall response following secondary influenza infection. We generated x31-immune animals in the presence or absence of NK cells and challenged the mice at memory (45–50 dpi) with the PR8 heterosubtypic strain of influenza which shares the internal x31 NP epitope but expresses distinct external epitopes/antibody targets. We hypothesized that having more Ag-specific memory cells positioned in the lung prior to challenge would result in a larger recall response in the animals depleted of NK cells in the primary response, resulting in enhanced protection and reduced viral titers after challenge.

**Figure 4:**
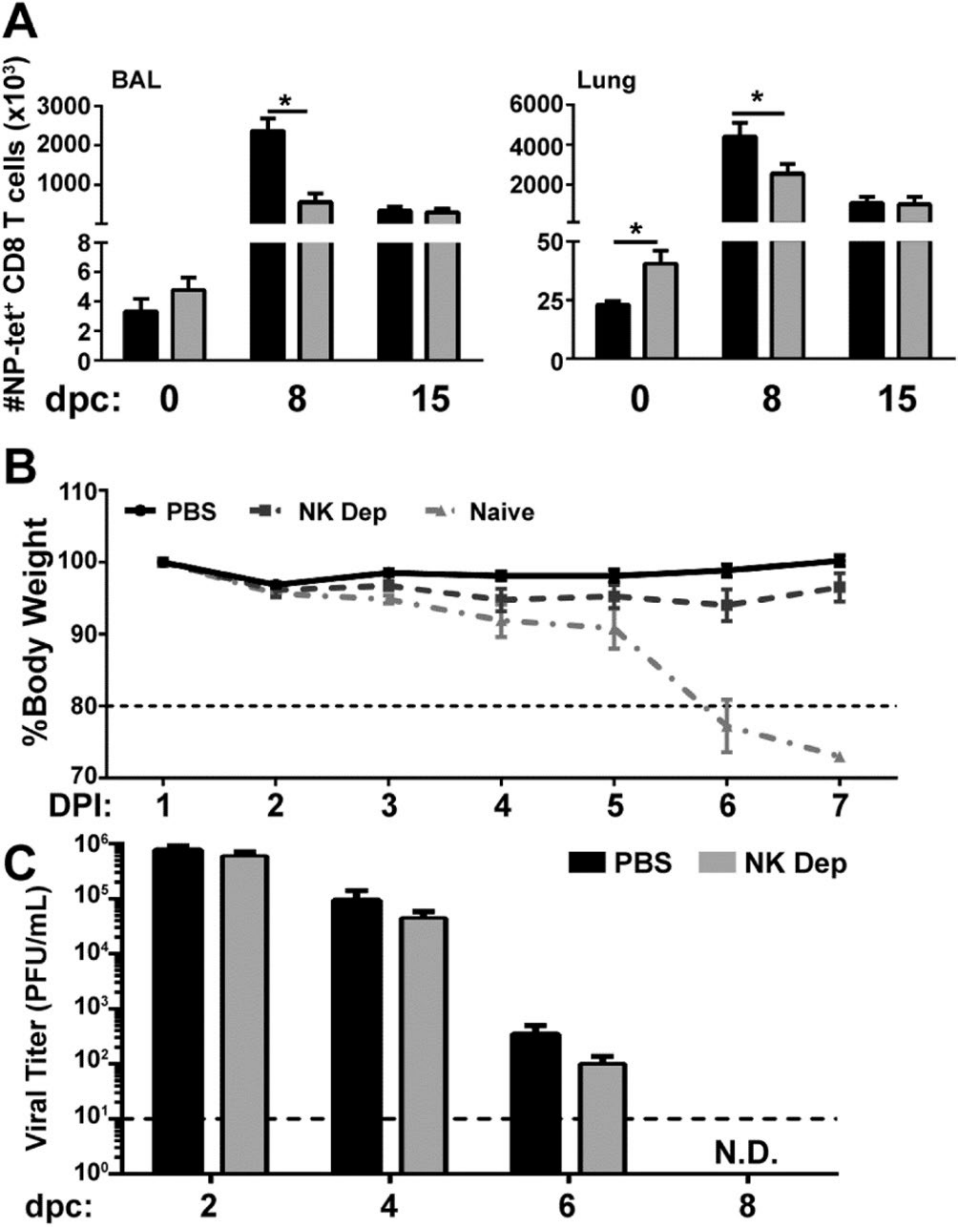
NK depleted animals are equally protected from heterosubtypic challenge by a numerically smaller CD8^+^ recall response compared to NK sufficient animals. C57BL/6 mice were infected with 10^3^ PFU x31 and administered anti-NK depleting antibody on −1, 1, 3 dpi to develop x31 immune mice. (**A**) The x31 immune mice were subsequently challenged with 10^5^ PFU PR8 50 dpi. The total number of CD44^hi^NP-tet^+^CD8^+^ cells ± SEM recovered from the BAL (left) and lung (right) of NK sufficient (black) and NK depleted (grey) mice was determined at 0 (45 dpi), 8, and 15 dpc. (**B**) The percent of body weight lost ± SEM by x31 immune mice generated in the presence (solid black) or absence (dashed, dark grey) of NK cells after a 10^5^ PFU PR8 challenge was compared to naïve mice (dash-dot, light grey) after a primary 500 PFU PR8 infection. Horizontal dashed line represents weight loss of 20%; the humane endpoint of 30% was reached by naïve controls on 7dpc. (**C**) The viral titer of lung homogenate recovered from NK sufficient (black) or depleted (grey) immune animals was measured 2, 4, 6 and 8 dpc (N.D. = not detectable). Horizontal dashed line represents the limit of assay detection. Graphs represent pooled data from 2 independent experiments using n = 5 mice/group/time point (total of 10 mice per group/time point). *p < 0.05; unpaired Student’s t-test with Holm-Sidak multiple comparisons correction.

Prior to challenge, we recovered nearly double the number of NP-specific CD8^+^ T cells from the lungs of NK-depleted mice. As we monitored the accumulation of the NP-specific memory CD8^+^ T cells in the BAL and lung at 8 and 15 days post-challenge (dpc), we recovered fewer NP-specific CD8^+^ T cells from these tissues in NK-depleted mice (nearly 1/3 and 1/2, respectively) at the proliferative peak of the recall response at 8 dpc (Fig. 4A). Despite a numerically reduced recall response, NK-depleted mice were equally protected after challenge as they demonstrated similar weight loss (Fig. 4B) and survival to NK-sufficient animals. While no immune mice succumbed to infection, naïve animals reached the humane endpoint of 30% weight loss by 7dpc (data not shown). While this enhanced recovery observed in NK-depleted animals could be attributed to the elevated levels of Ag-specific CD8^+^ T_RM_ cells in the lung at the time of viral challenge, it is possible that reduced NK-derived inflammation from the protracted depletion of NK cells from the lung in NK-depleted mice (Supplementary Fig. S2) could also curtail infiltration and expansion of Ag-specific CD8^+^ T cells following viral re-challenge. Nonetheless, viral titers were decreased in NK-depleted animals at 2, 4 and 6 dpc, albeit not statistically significant (Fig. 4C). Moreover, there was no observed difference between the groups regarding time to viral clearance. Therefore, our data suggest that CD8^+^ T_mem_ generated in the absence of NK cells may be functionally superior upon recall when compared to their NK-sufficient counterparts, as they do not require either in situ proliferation or tissue recruitment to amass a large number of new CD8^+^ T effectors to reduce viral load.

**Figure 5:**
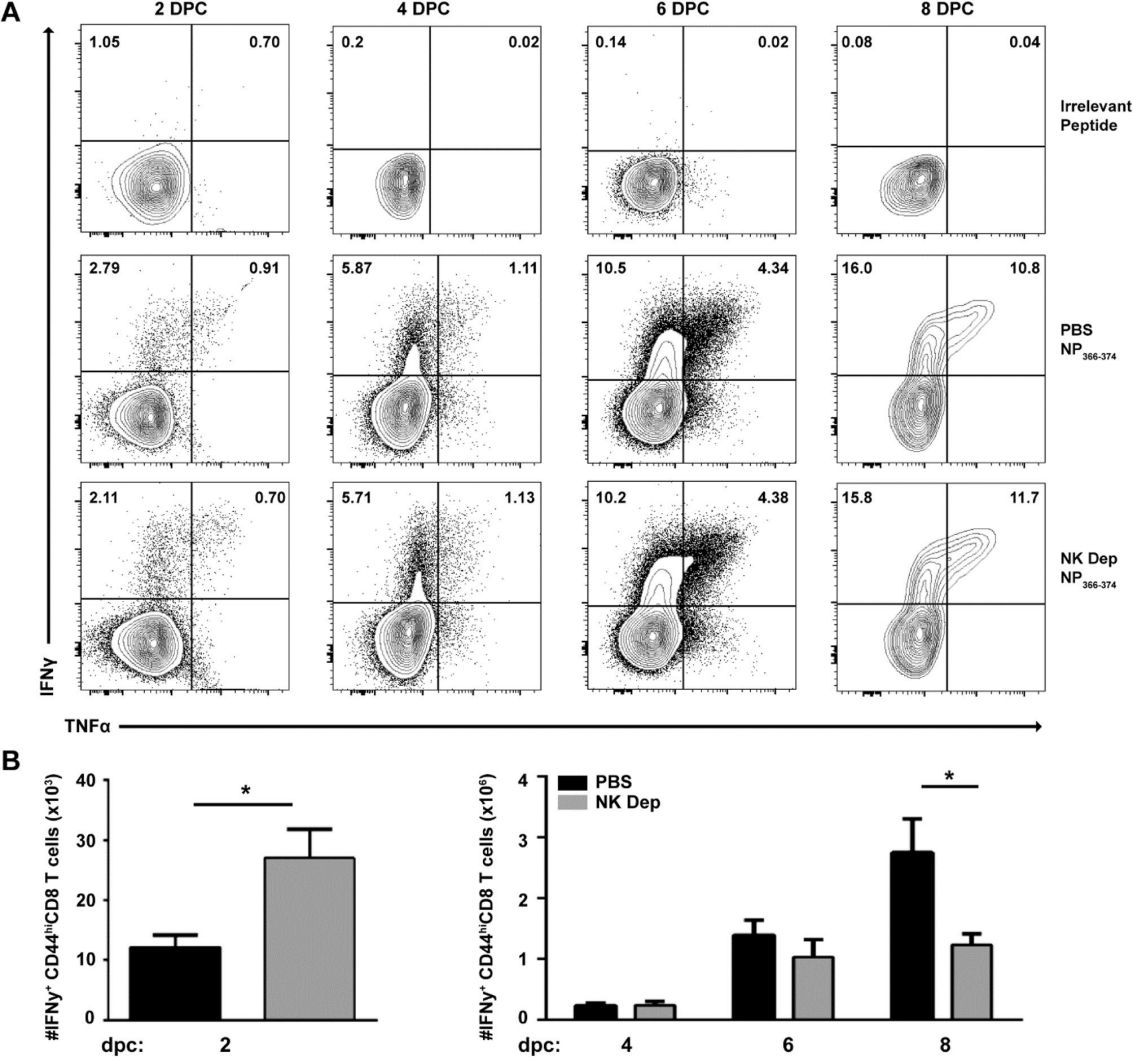
CD8^+^ T_mem_ generated in the presence and absence of NK cells produce equivalent percentages but different overall amounts of IFN-γ over time x31 immune mice generated in NK-sufficient or deficient conditions were challenged with 10^5^ pfu PR8 and lymphocytes were recovered from the lung at the indicated dpc. (**A**) Following re-stimulation with the indicated peptide, TNF-α and IFN-γ was detected by intracellular staining as depicted in the representative dot plots. (**B**) Absolute number of IFN-γ^+^ CD8^+^ T cells recovered from lung of NK-deficient (grey) or sufficient (black) mice on indicated dpc. Graphs represent pooled data from 2 independent experiments using n = 5 mice/group/time point (total of 10 mice/ time point*p < 0.05; unpaired Student’s t-test with Holm-Sidak multiple comparisons correction.

To assess the functionality of CD8^+^ T_mem_ developed in the absence of NK cells, we generated × 31 immune mice in the presence or absence of NK cells and challenged with PR8 as previously described. CD8^+^ T cells were subsequently recovered from the lung at 2, 4, 6, and 8 dpc, re-stimulated ex vivo with the influenza NP_366-374_ peptide or irrelevant peptide and assessed for production of IFN-γ and TNF-α, as airway resident CD8^+^ T cells are capable of curtailing viral replication through production of these cytokines^35^. CD8^+^ T_mem_ recovered from the lung of NK-depleted and sufficient mice harbored equivalent proportions of IFN-γ and TNF-α producing cells (Fig. 5A) and equivalent production of these cytokines on a per cell basis (data not shown). However, when comparing the kinetics of the overall total number of cytokine producing cells, the total number of IFN-γ^+^ cells in NK-depleted animals peaked earlier and with less overall cellular accumulation, resulting in ~ 1/2 the number of IFN-γ^+^ CD8^+^ T cells in the lung at 8 dpc compared to NK-sufficient animals. There was no difference in the total number of IFN-γ^+^ cells between PBS or isotype treated controls (data not shown). Interestingly nearly 2 × more IFN-γ^+^ CD8^+^ T cells were recovered from the lung of NK-depleted vs PBS or isotype control treated animals (Fig. 5B, and data not shown) at 2dpc. Studies by Wu et al*.* demonstrated that infiltration of reactivated peripheral CD8^+^ T_mem_ does not occur before 3 dpc^5^, therefore the IFN-γ^+^ CD8^+^ T cells are likely T_RM_ reactivated rapidly in situ. Together, these data suggest that ablation of NK cells during primary immune response to influenza allows for an equally functional, protective recall response with fewer Ag-specific CD8^+^ T cells entering the lung and lung airways after challenge. We postulate that this could be attributed to the increased number of lung T_RM_ (Fig. 2B) which are quickly activated and converted to secondary effector cells producing significant levels of local IFN-γ; however, we cannot completely rule out possible contributions from the protracted depletion of lung specific NK cells (Supplementary Fig. S2) in our depletion model.

### CD8^+^ T_mem_ formation in the lung is independent of NK-cell derived IFN-γ

Since NK cell depletion increases the number of NP-specific CD8^+^ T_RM_, we next sought to determine the mechanism by which NK cells impair anti-viral CD8^+^ T_RM_ development. Previous studies demonstrated that NK cells can influence T cells directly via lysis, inhibiting their proliferation, and controlling viral load^16,19,20^. However, NK cells are also potent producers of IFN-γ which can contribute to CD8^+^ T cell activation or promote contraction by increasing pro-apoptotic factors^36–39^. Additionally, IFN-γ can block the development of CD127 expressing memory precursor CD8^+^ T cells if induced during or shortly after T cell priming in a DC immunization model^9^. Thus, loss of NK-derived IFN-γ could explain the numeric differences in the anti-viral CD8^+^ T cells observed in NK-depleted versus sufficient mice.

**Figure 6:**
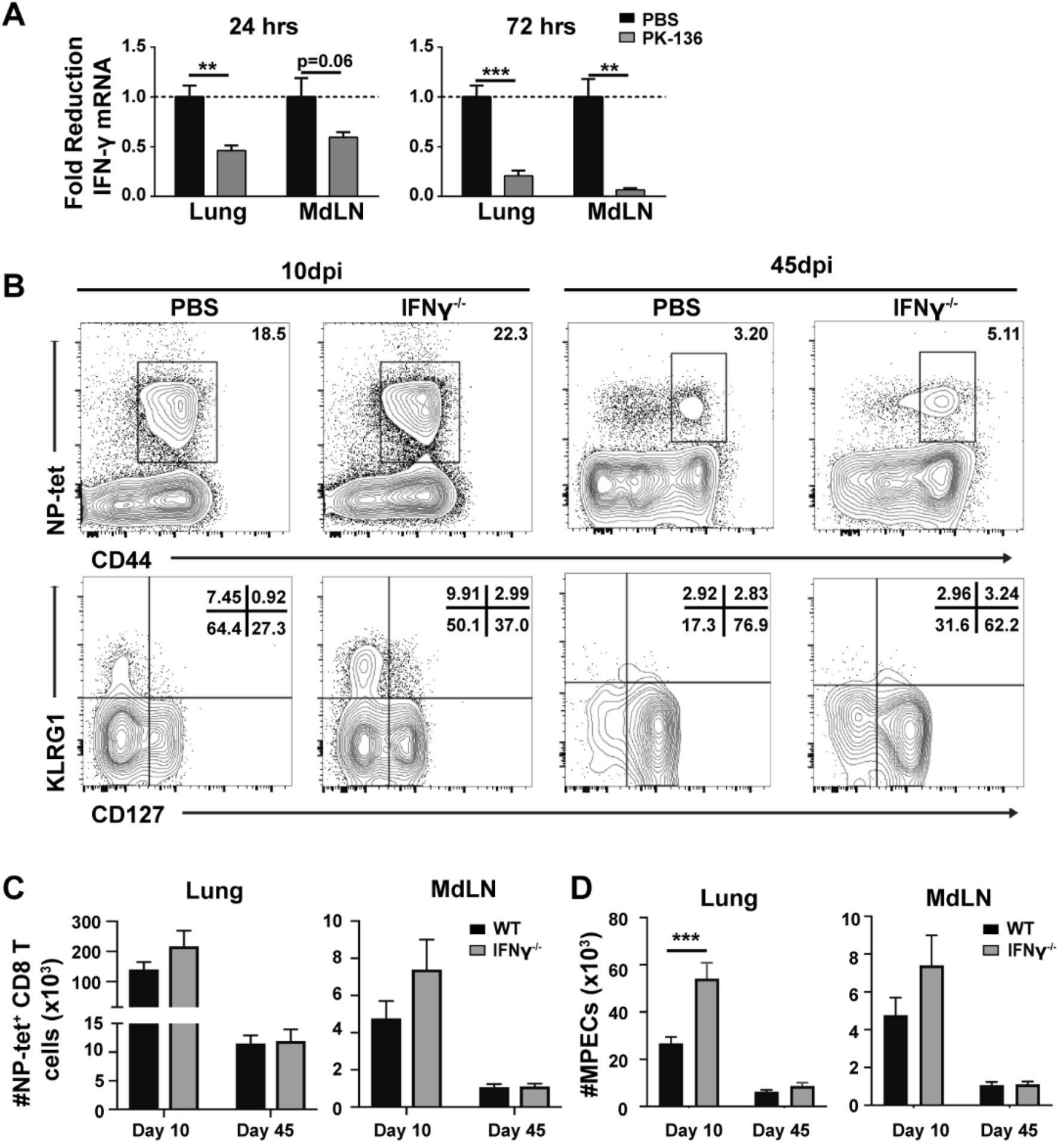
IFN-γ deficiency enhances the total number of memory precursors, but not T_mem_ following viral respiratory infection. C57BL/6 mice were infected with 10^3^ PFU x31 and administered the NK depleting antibody on −1, 1, and 3 dpi. (**A**) The relative expression of IFN-γ transcript recovered from bulk lung tissue and MdLN of NK-sufficient (black) and depleted (grey) mice 24 and 72 h post infection. (**B**) C57/BL6 WT and IFN-γ^−/−^ mice were infected with 10^3^pfu x31, sacrificed 10 and 45dpi and frequency of CD8^+^CD44^+^NP-tet^+^ cells as well as subsequent CD127 and KLRG1 expression assessed. (**C**) Total number ± SEM of activated CD44^+^ NP-tet^+^ CD8^+^ cells in WT (black bar) and IFN-γ^−/−^ (grey bar) mice. (**D**) Total number of CD127^+^KLRG1^-^ MPECs in the lung and MdLN of WT (black) and IFN-γ^−/−^ (grey) mice 10 and 45dpi is depicted. Graphs are representative of data from 2 independent experiments using n ≥ 3 mice/group/time point (total 6 animals per group/time point). *p < 0.05, **p < 0.01, ***p < 0.001; unpaired Student’s t-test with Welch’s correction (for mRNA analysis).

To determine the contribution of NK-derived IFN-γ to our influenza model, we measured IFN-γ transcripts in the lung and MdLN of NK-depleted and sufficient mice prior to the activation (48–72 h) and arrival (72–96 h) of T cells in the lung^40,41^. As expected, NK-depleted mice expressed IFN-γ transcript at lower levels, approximately one half and one quarter of that observed in the lung of NK-sufficient mice at 24 and 72 h post infection, respectively (Fig. 6A). Similarly, NK-depleted mice expressed approximately one half and one tenth IFN-γ transcript levels of NK-sufficient mice in the MdLN at 24 and 72 h post infection, respectively (Fig. 6A). We next examined the development of NP-specific memory precursors and T_mem_ at 10 and 45dpi in both WT and IFN-γ^−/−^ mice (Fig. 6B). While we observed no significant difference in the total number of NP-specific CD8^+^ T cells in the lung or MdLN of WT or IFN-γ^−/−^ mice 10dpi (Fig. 6C, left), IFN-γ^−/−^ mice harbored an increased frequency of CD127^+^ MPECs (Fig. 6B), which translated into an increase in total number of lung MPECs (Fig. 6D, left). By 45dpi this numerical advantage in MPECs was lost, and equivalent levels of NP-specific CD8^+^ T cells were recovered from both the lung and mdLN of WT and IFN-γ^−/−^ mice (Fig. 6C), with comparable expression of CD127 (Fig. 6B,D). This indicates that while IFN-γ may play a role in the development of memory precursors through suppressing CD127 expression on influenza-specific CD8^+^ T cells, it does not alter the number of NP-specific CD8^+^ T cells in the memory pool. Thus, NK cell-derived IFN-γ may be one of multiple mechanisms by which NK cells regulate memory CD8^+^ T cell development in the lung.

**Figure 7:**
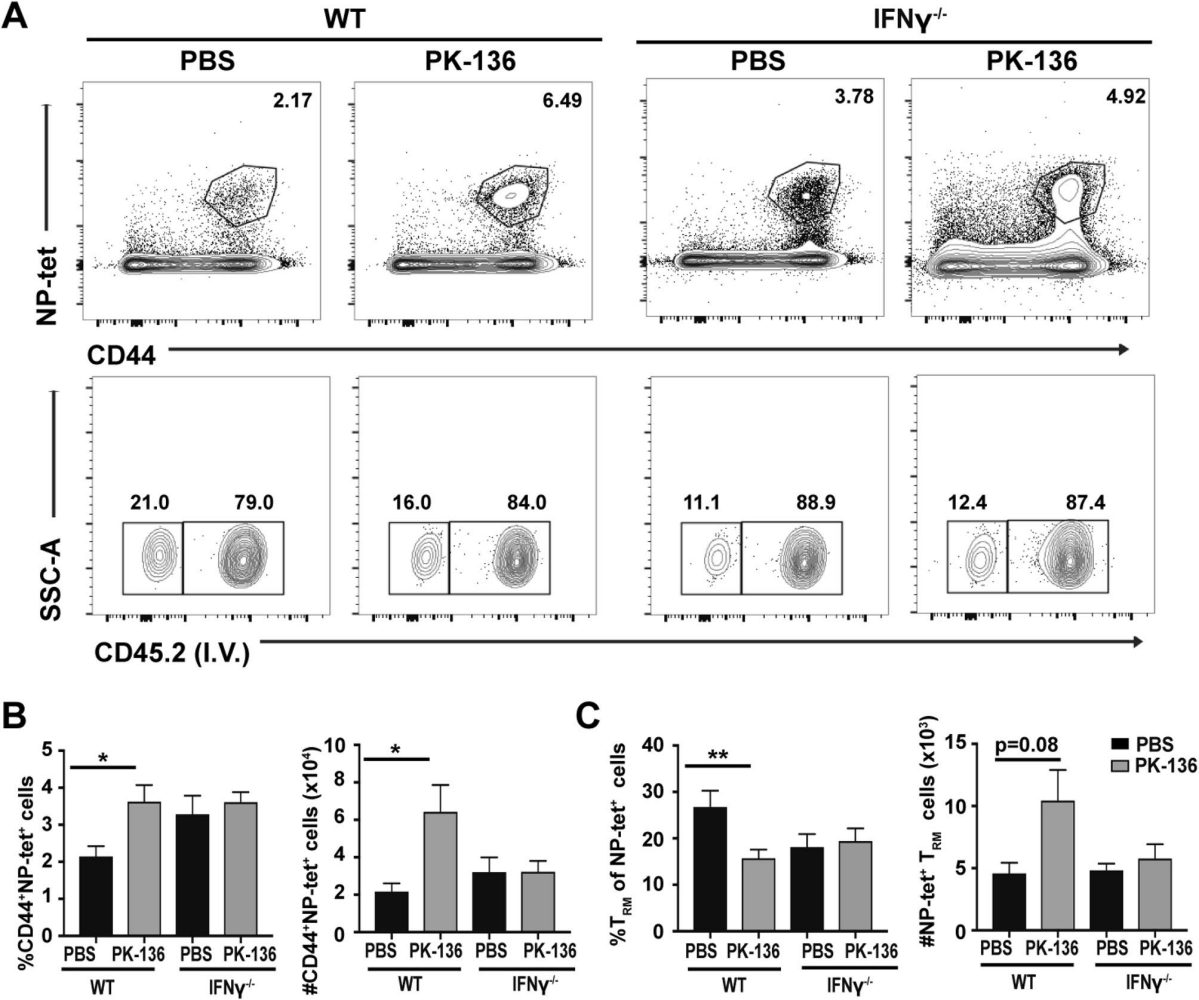
Enhanced respiratory T_RM_ development in NK-deficient mice is independent of IFN-γ. (**A**) C57/BL6 WT and IFN-γ^−/−^ mice were administered 200ug of PK136 or 200ul PBS 1 day prior to infection with 10^3^pfu x31. Mice were sacrificed 35 days later and frequency of CD8^+^CD44^+^NP-tet^+^ cells, as well as NP-tet^+^ T_RM_ cells (identified by lack of staining with an anti-CD45 antibody administered i.v. 3 min prior to euthanasia) in the lung assessed. Frequency and total number ± SEM of (**B**) CD8^+^CD44^+^NP-tet^+^ cells and (**C**) NP-tet^+^ T_RM_ cells in the lung 35 days post infection in WT or IFN-γ^−/−^ NK-depleted (grey bar) and PBS treated (black bar) mice. Graphs are representative of pooled data from 2 independent experiments using n ≥ 3 mice/group (total WT/PBS: n = 7, WT/NKdep: n = 11, IFN-γ^−/−^/PBS: n = 6, IFN-γ^−/−^/PK-136: n = 8 depicted). *p < 0.05, **p < 0.01; One-way ANOVA with multiple comparisons correction.

To further explore whether NK cell-derived IFN-γ contributes the development of CD8^+^ T_RM_ after influenza infection, we examined the NP-specific T_RM_ cells present in the lung 35 dpi in NK-sufficient or deficient WT and IFN-γ^−/−^ mice. We observed an increase in the frequency and total number of NP-specific CD8^+^ T cells recovered from the lungs of NK-depleted WT mice, which was not observed in IFN-γ^−/−^ mice irrespective of NK-depletion (Fig. 7A,B). While more NP-specific T_RM_ cells were recovered from the lungs of NK-deficient WT mice (although not statistically significant) compared to NK-sufficient WT controls (Fig. 7C), elevated T_RM_ numbers were again not observed in the lungs of IFN-γ^−/−^ mice, regardless of NK depletion (Fig. 7C), indicating that T_RM_ formation in the lung following influenza infection occurs independent of both global and NK cell-derived IFN-γ. Together, these data suggest that NK cells regulate T_RM_ development in the respiratory tract, albeit independent of IFN-γ production. While the mechanism by which NK cells regulate T_RM_ formation within the lung is still unknown, reducing NK cell activation during CD8 T cell priming may provide a mechanism to enhance regional CD8^+^ T cell protection with a reduced potential for immunopathology.

## Discussion

Diversity in the CD8^+^ T_mem_ lineage is imprinted by direct cellular encounters and soluble stimuli, beginning as early as the initial priming event in a regional draining lymph node and continuing within the infected tissue where residency may be established. While many signaling pathways have been identified which bias antigen-specific CD8^+^ T cells towards an effector or T_mem_ fate^42^, less is known about the signals responsible for imprinting the T_RM_ program. In this study, we examined the net contribution of NK cells to the subsequent generation of anti-influenza CD8^+^ T_mem_ subsets. We found that NK cell depletion during influenza infection results in numerically increased CD8^+^ T_mem_ (Fig. 1B) akin to what has been reported after systemic lymphocytic choriomeningitis virus (LCMV) infection^15^. NK depletion enhanced the numbers of NP-specific T_mem_ in the lung 45 days following influenza infection, which remained elevated in both the lung and lung draining MdLN out to 90 days post infection (Fig. 1A). Moreover, similar to LCMV infection^15^, NK depletion resulted in NP-specific cells had enhanced expression of the IL-7R alpha subunit (CD127), which is known to bolster CD8^+^ T memory formation by promoting survival through the contraction phase of the immune response^26,27^. In fact, increased expression of CD127 in NK-depleted mice was correlated elevated numbers of lung MPECs 45dpi (Fig. 1D), suggesting that NK depletion may enhance the survival of lung T_mem_ following respiratory infection.

Subsequent analysis of the NP-specific cells in the lung revealed that while all T_mem_ populations including T_EM_ were numerically enhanced (data not shown), NK-depleted animals harbored 2 × more tissue-embedded T_RM_ compared to their NK-sufficient counterparts (Fig. 2C), implicating NK cells as regulators of the T_RM_ program. Additionally, the CD8^+^ T_mem_ pool in the respiratory tract of animals depleted of NK cells in the primary infection was equally protective against heterosubtypic challenge compared to control animals (Fig. 4B). While the proportion of reactivated CD8^+^ T_mem_ recovered from the lung of NK-depleted or sufficient animals producing IFN-γ and TNF-α on days 2, 4, 6 and 8 post challenge (Fig. 5A) was equivalent, the overall number of lung CD8^+^ T cells producing IFN-γ significantly increased 2 dpc in the animals depleted of NK cells in the primary (Fig. 5B). Given that T_RM_ cells produce significant IFN-γ in the lung prior to the recruitment of reactivated peripheral T_mem_ and the known contribution of CD8^+^ T_RM_ cells in protection against heterosubtypic influenza infection^5^, our data suggests that positioning more CD8^+^ T_RM_ cells at the site of infection can control viral titers without the need for reactivation of peripheral T_mem_ cells. Indeed, animals depleted of NK cells in the primary infection possessed lower challenge viral titers compared to NK-intact animals at all times examined (2, 4, and 6 dpc) (Fig. 4C), albeit not to statistically significant levels. Therefore, a threshold pool of T_RM_ was reached in NK-depleted animals which sufficiently reduced antigen/inflammation to levels bypassing the need for reactivation and proliferation (Fig. 4A) of peripheral T_mem_ upon viral challenge. This is relevant considering that CD8^+^ T cell-mediated tissue damage during clearance of influenza infection may contribute to secondary bacterial infection and increased mortality. Therefore, limiting T cell proliferation after reactivation could ameliorate these undesirable consequences.

Studies have shown that adoptive transfer of in vitro-derived memory CD4^+^ T cells into NK-depleted BALB/c mice curtailed influenza-induced morbidity via reduced NK-driven inflammation^43^. In our studies, NK cells remained dramatically reduced in the lung at the time of viral re-challenge (Supplementary Fig. S2). It is therefore possible that the improved animal recovery observed in the adoptive transfer study may be partially due to the protracted depletion of lung NK cells. Unlike McKinstry et al^43^, we did not observe a significant difference in weight loss or recovery between NK-sufficient and NK-deficient animals following viral challenge (Fig. 4B). Instead, we observed an early increase in the total number of Ag-specific, IFN-γ producing CD8^+^ T cells and reduced recruitment of secondary CD8^+^ effector T cells at later time points (Fig. 5) in NK-depleted mice, the latter which could also be attributed to reduced inflammation. Thus, while differences in experimental methodology may account for some of the observed discrepancies between these studies, cumulatively both sets of data suggest that NK cell depletion during influenza challenge likely alters the host environment and response to infection.

NK cells can modulate the anti-influenza CD8^+^ T cell response in a variety of ways, through suppression of recently activated CD8^+^ T cells^14,15^, direct elimination of infected CD8^+^ T cells^13,16–18,44^, or through elimination of viral reservoirs, impacting the overall magnitude of CD8^+^ T cell priming. Additionally, NK cell-mediated culling of activated CD4^+^ helper T cells limited effector CD8^+^ T cell proliferation, resulting in numerically reduced memory^15^. However, we did not observe significantly more CD8^+^ T cells in NK-depleted mice at the proliferative peak following influenza infection (Fig. 1B), suggesting an alternate NK cell-mediated mechanism was responsible for the increased number of CD8^+^ T_mem_. NK-DC crosstalk^45,46^ is not likely affected in our model as the number of DCs and their surface expression of MHC-I, MHC-II and CD80 at 1–5 dpi was equivalent in NK-depleted and sufficient mice (data not shown). Thus, the increased number of CD8^+^ T_RM_ we observed in NK-depleted mice cannot be ascribed to bystander regulation of DCs known to impact CD8^+^ T_RM_, suggesting that NK cells directly regulate CD8^+^ T_RM_ development. Recent studies demonstrated that NK cells can directly eliminate activated, virus-specific, CD8^+^ T cells via their natural cytotoxicity receptor (NCR1) during acute LCMV infection^47^. Absence of NCR1 results in a more robust CD8^+^ T cell response, accompanied by increased immunopathology^47^, suggesting that not only can NK cells directly control the virus-specific CD8^+^ T cell pool, but this regulation may be critical for preserving tissue integrity, which would be of particular importance in vital organs such as the lung.

In our system, a single injection of PK136 mAb depletes NK cells for 4 wks. It is of note that CD8^+^ T cells can upregulate NK1.1 during influenza virus infection^48,49^ and may be depleted during anti-NK1.1 treatment. However, we did not observe differences in influenza-specific CD8^+^ T cell numbers between NK-depleted or control depleted at day 10 or 36 post-infection (data not shown). While virus-specific CD8^+^ T cells did express low levels of NK1.1, expression was almost tenfold lower than that of NK cells (data not shown) potentially preventing NK1.1-mediated depletion. Furthermore this depletion scheme has the potential to deplete both NK1.1^+^ NKT cells^24^ and ILC-1s^25^. We determined that NKT cells (which do not largely expand or become activated in our model of influenza infection (Supplementary Fig. S1)) likely do not play a large role in respiratory T_RM_ formation. However, due to their phenotypic similarities to NK cells, we were unable to directly assess the presence of type I ILC following influenza infection and thus cannot eliminate their potential contribution to our study. However, functionally NK cells would be quite relevant in modulating CD8^+^ T cell responses if their activation or inhibition signals are perturbed. While the NK inhibitory signaling via MHC I expression is not compromised by influenza infection^50^, expression of the NKp46 ligand hemagglutinin (HA) and other stress ligands would be limited to 10 dpi (by which point the x31 virus is cleared^51^). Additionally, presence of NK cell activating cytokines IL-15, type I IFN and IL-12 also cease with inflammation by 15 dpi^42,52–54^, limiting the effects of NK cells on CD8^+^ T cell immunity to within this time frame. Depletion of NK cells during CD8^+^ T cell contraction had no effect on T_mem_ formation; however, NK depletion during CD8^+^ T cell activation/proliferation and migration enhances T_mem_ development (Fig. 3D). It is also during this early time frame (< 10 dpi) where the highest levels of NK cell activation is observed, indicating that NK cell regulation of T_mem_ formation occurs coordinate with high levels of NK cell activation.

To elucidate the mechanism by which NK cells regulate respiratory T_RM_ formation, we focused our attention on IFN-γ, as activated NK cells are potent producers of IFN-γ which can antagonize CD8^+^ T_mem_ development when present during or shortly after priming^9,55^. In fact, CD127 expression, which is induced via an IL-10/mTOR pathway^56^ downstream of STAT3, can be modulated by IFN-γ-mediated suppression of IL-10 or dephosphorylation of STAT3^57^. Moreover, anti-influenza CD8^+^ T_mem_ are enhanced in both IFN-γ^−/−^ and IFN-γR1^−/−^ mice and this is directly attributed to modified CD127 and Bcl-2 expression^58^. We also observed an increased number of CD127^hi^ NP-specific CD8^+^ T cells in the lungs of NK-depleted mice 45 dpi (Fig. 1D), suggesting that NK-derived IFN-γ could regulate CD127 responsiveness and subsequent development and survival of T_mem._ If loss of IFN-γ alone was responsible for the elevated CD8^+^ T_RM_ observed within the lung of NK-depleted mice, then this observation should be replicated in IFN-γ^−/−^ mice. However, we observed no difference in the total number of NP-specific T_mem_ generated in the lung or dLN at 45 dpi in IFN-γ^−/−^ animals (Fig. 6C) as well as no difference in the total number of lung T_RM_ 35dpi (Fig. 7B,C), indicating that T_mem_ (and furthermore T_RM_) generation in the lung following influenza infection may be in part IFN-γ independent. However, surprisingly, IFN-γ^−/−^ mice depleted of NK cells do not display enhanced numbers of lung T_RM_ observed in wild-type NK-depleted mice (Fig. 7), which would suggest an IFN-γ dependent mechanism is involved. Thus, there appears to be an additional NK-independent, IFN-γ-dependent pathway present to regulate T_RM_ formation within the lung. This could be attributed to the mechanism of T_RM_ regulation by IFN-γ, through either STAT3 (or IL-10/mTOR) dependent or independent pathways; however, we observed no difference in IL-10 transcripts between NK-depleted and sufficient animals (data not shown). However, we cannot eliminate the possibility that there is another IFN-γ producing population (possibly CD4^+^ T cells, DCs, or alveolar macrophages) which contributes to T_RM_ formation within the lung. While the specific mechanism by which NK cells regulate T_RM_ formation remains elusive, future studies will attempt to dissociate the specific interactions and/or soluble mediators responsible for modulating T_RM_ programming including IFN-γ independent regulation of STAT3 and the T_RM_ associated transcription factor Hobit.

In summary, our data suggest that curtailing NK activation during initial infection or vaccination could generate sufficient, cross-protective CD8^+^ T_RM_ with limited need for proliferation after reinfection. While ablation of NK cells during vaccination is impractical, influenza viruses can stimulate NK cells via interaction between the viral hemagglutinin and the NK cell activating receptor NKp46^59^. Thus, it may be possible to limit or differentially modulate NKp46 receptor ligation and resultant NK cell activation during vaccination with live attenuated viruses in order to enhance protective CD8^+^ T_RM_ development, limiting respiratory immunopathology and susceptibility to secondary bacterial infection after re-challenge.

## Methods

### Mice, viruses, infections and NK cell depletion

All experiments involving infectious virus were performed under animal biosafety level 2 (ABSL2) conditions. Age and sex matched C57BL/6 WT (Charles River (Wilmington, MA)) and IFN-γ^−/−^ mice (generously provided by Dr. Rick Tarleton) were anesthetized using 2, 2, 2-Tribromoethanol and infected intranasally (i.n.) with 10^3^ pfu influenza A/HK- x31 (x31, H3N2), 500 pfu A/PR8 (PR8, H1N1) or 10^5^ pfu PR8 in 50 μl PBS. Where noted, NK cells were depleted via intravenous (i.v.) injection of 200 µg of anti-NK1.1 antibody (clone PK136; BioXcell, West Lebanon, NH) or Isotype control (IgG2a clone C1.18.4; BioXcell) in 200µL sterile PBS every other day for 5 days starting 1 day prior to infection, unless otherwise specified. All animal work and experimental procedures presented here were approved by the Institutional Animal Care and Use Committee of University of Georgia (AUP A2018 05–004-Y2-A2), and conducted in compliance to the relevant regulations and guidelines, including those in the ARRIVE checklist.

### Quantitative RT-PCR

Sample collection and preparation for quantitative PCR was conducted as previously described^52^. Lungs were collected and RNA purified from the samples using the RNeasy Plus Mini Kit (Qiagen, Valencia, CA). Reverse transcriptions were performed using the High Capacity cDNA Reverse Transcription Kit from Applied Biosystems (Foster City, CA). Quantitative PCR assays were prepared using the ABI TaqMan Gene Expression Master Mix from ABI 7500 Real Time PCR System (Applied Biosystems, Grand Island, NY). Quantitative real-time RT-PCR was performed using TaqMan technology using Ifn-γ-FAM (Mm01168134_m1) and 18 s-VIC (#4319413E) assays from Life Technologies (Grand Island, NY) in single-plex reactions and assessed on a 7500 Real Time PCR System (Applied Biosystems, Grand Island, NY). Thermal cycling conditions were 2 min at 50 °C, 10 min at 95 °C, and 45 cycles of denaturation (95 °C for 15 s) and annealing (60 °C for 60 s).

### Plaque assays

Plaque assays were performed as previously described^60^. Briefly, whole lungs were extracted and homogenized using a Tissue Lyser (Qiagen, Hilden, Germany). Serial dilutions of 10% homogenate were incubated atop confluent monolayers of Madin-Darby canine kidney cells grown in 12 well plates at 37 °C. One hour later, cell layers were washed and overlaid with MEM containing 1.2% Avicel microcrystalline cellulose (FMC BioPolymer, Philadelphia, PA). After 72 h, the overlay was removed and the cells were washed, fixed with cold methanol/acetone (60:40%) and stained with crystal violet. Plaques were counted and plaque-forming units per mL of lung homogenate determined.

### Tissue preparation and flow cytometry

Single cell suspensions from tissues were obtained as previously described^52^. Lung airway cells were obtained by bronchoalveolar lavage (BAL) whereby 1 ml of PBS is introduced and recovered from the lung airway 3 × after tracheal intubation. Subsequently, perfused lungs were excised, minced and incubated with 1.25 mM EDTA at 37 °C for 30 min followed by incubation with 150 units/mL of collagenase (Life Technologies, Grand Island, NY) for 1 h. After passage through cell strainers, lymphocytes were resuspended in 44% Percoll underlaid with 67% Percoll, centrifuged, and the cellular interface collected. Lymph nodes and spleen were mechanically disrupted, passed through cell strainers, and erythrocyte depleted using Tris-buffered ammonium chloride. Cells were enumerated using a Z2 Coulter Particle Counter (Beckman Coulter, Brea, CA). Intravascular staining was performed by injecting mice with 3 µg FITC-conjugated anti-CD45.2 (104) i.v. three minutes prior to euthanasia. Influenza nucleoprotein (NP) MHC class I [H-2D(b)/ASNENMETM] tetramers were generated at the National Institute of Allergy and Infectious Diseases Tetramer Facility (Emory University, Atlanta, GA) and used for surface staining at RT for 1 h with a combination of other mAbs (clones): PerCP-Cy5.5-conjugated anti-CD8α (53–6.7), anti-NKp46 (9E2), or anti-CD69 (H1.2F3), FITC-conjugated anti-CD44 (IM7) oranti-NKp46 (29A1.4), PE-conjugated anti-CD127 (A7R34), anti-NKp46 (29A1.4), anti-NK1.1 (PK136), or anti-CD103 (2E7), APC/Cy7-conjugated anti-CD62L (MEL-14), anti-CD8α (53–6.7), or anti-CD127 (A7R34), PE/Cy7-conjugated anti-KLRG1 (2F1), or anti-CD8α (53–6.7), APC-conjugated anti-CD3 (145-2C11), for 20 min at 4 °C without tetramer. All antibodies in this study were purchased from eBioscience or Tonbo Biosciences (both San Diego, CA). Data was acquired using a LSR II with FacsDiva software (BD Biosciences, San Jose, CA) and analyzed using FlowJo software (Tree Star INC, Ashland, OR).

### CD8 T cell re-stimulation assay

Lymphocytes were incubated at 37 °C for 5 h in the presence of influenza NP_366-374_ (ASNENMETM) or irrelevant ovalbumin (OVA)_257–264_ (SIINFEKL) peptide in the presence of GolgiStop (BD Pharmingen, San Diego, CA). Subsequently, cells were stained with anti-CD8α (53–6.7) and anti-CD44 (IM7) mAbs for 20 min at 4 °C and fixed in 2% paraformaldehyde overnight. The next day, samples were permeabilized with Perm/Wash (BD Biosciences, San Diego, CA), stained with FITC-conjugated anti-IFN-γ and PerCP5.5-conjugated anti-TNFα for 30 min at 4 °C and analyzed by flow cytometry.

### Statistics

Statistical analysis was carried out using GraphPad Prism software (GraphPad Software, La Jolla, CA) and reviewed by a biostatistician. Significance was determined using Student’s two-tailed t-test (and Holm–Sidak multiple comparisons correction or Welch’s correction when appropriate) where the p-value was p < 0.05. All statistically significant results are reported.

## Supplementary Information

Supplementary Information
