# Supplementary material for: Enhanced generation of influenza-specific tissue resident memory CD8 T cells in NK-depleted mice
Source: Sci Rep. 2021 Apr 26;11:8969. doi: 10.1038/s41598-021-88268-7 (PMC8076325; doi:10.1038/s41598-021-88268-7)
Supplement: Supplementary file 1 — Supplementary Information [file 41598_2021_88268_MOESM1_ESM.pdf]

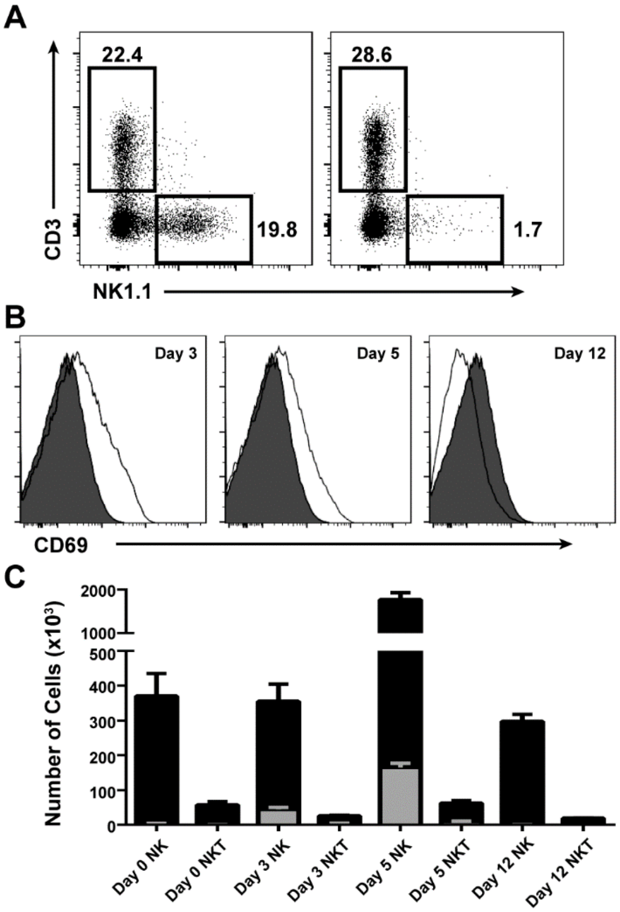

Supplemental Figure 1. Activation and expansion of NK cells following influenza infection. C57BL/6 mice were infected with 10^3 PFU x31 and administered anti-NK depleting antibody on -1, 1 and 3 dpi. (A) To assess depletion efficiency, the frequency of NK cells was determined in the lung of NK sufficient (left) and NK depleted (right) mice 5 dpi. (B) NK cells recovered from lung tissue of naive (solid histogram) and x31 infected (open histogram) mice on 3, 5, and 12 dpi were analyzed for expression of the activation marker CD69. (C) To examine the kinetics of the NK and NKT cell expansion and activation, NK ($CD3^-NK1.1^+NKp46^+$) and NKT ($CD3^+mCD1d^+$) cells were recovered from the lung of x31 infected mice at 0, 3, 5, and 12 dpi and their total number (black) and the number of those cells expressing CD69 (grey) ±SEM were determined. Data are representative of 3 independent experiments using n = 3 mice/group.

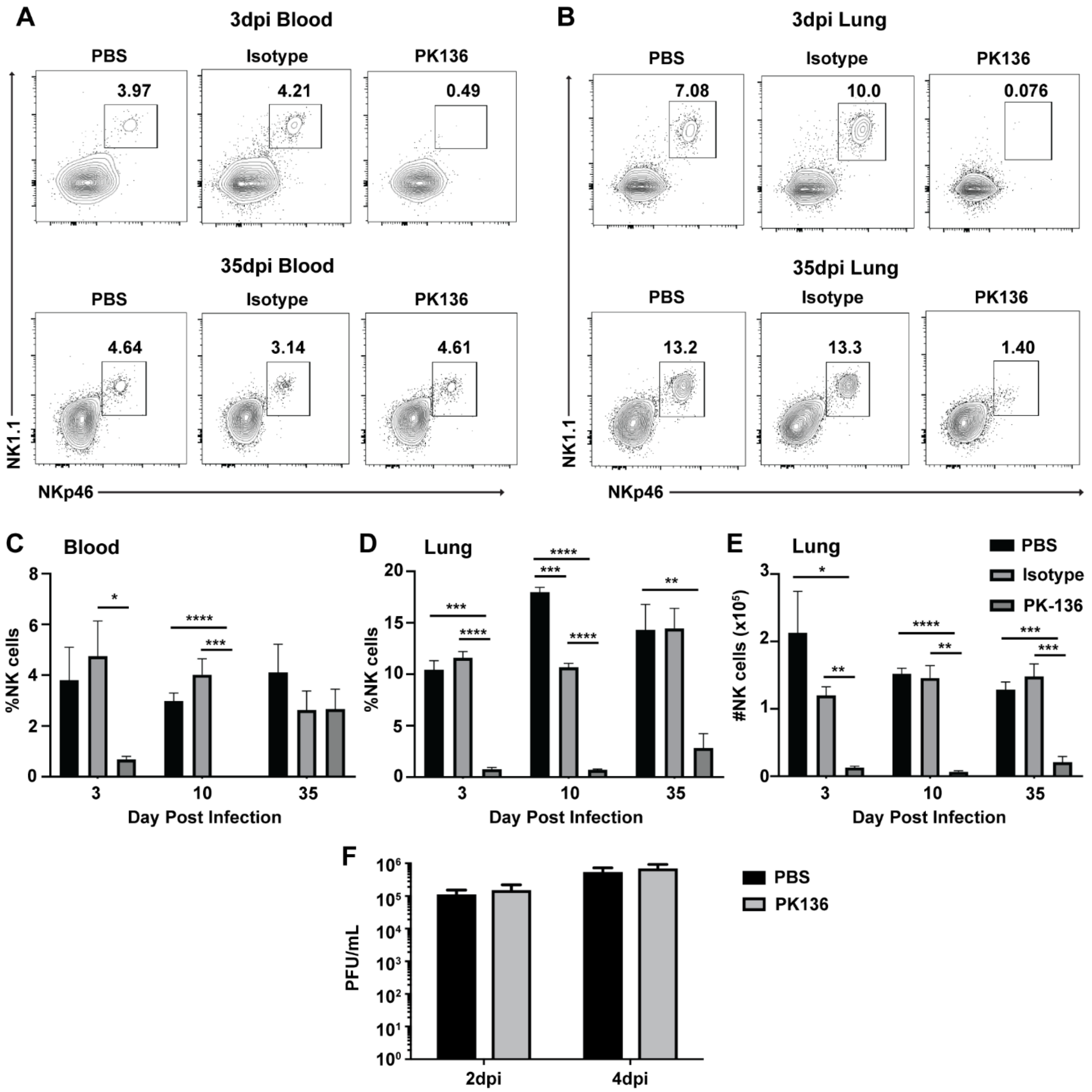
A
3dpi Blood
PBS
Isotype
PK136
3.97
4.21
0.49
35dpi Blood
4.64
3.14
4.61
NK1.1
NKp46
B
3dpi Lung
7.08
10.0
0.076
35dpi Lung
13.2
13.3
1.40
C
Blood
%NK cells
Day Post Infection
D
Lung
%NK cells
E
#NK cells (x10^5)
PBS
Isotype
PK-136
F
PFU/mL
2dpi
4dpi
PBS
PK136

Supplemental Figure 2. Administration of the PK136 mAb depletes NK cells for 4 weeks during an influenza infection. C57BL/6 mice were infected with 10^3 PFU x31 and administered 200ug anti-NK depleting antibody, isotype control or PBS on -1, 1 and 3 dpi. The frequency and total number of NK cells was determined in the blood and lung until the recovered NK cells in the blood of depleted animals was equivalent to homeostatic NK cell numbers in wild type mice. Representative flow of $NK1.1^+NKp46^+$ NK cells in the blood (A) and lung (B) of PBS treated, isotype treated or depleted (PK136 treated) mice 3dpi (top row) and 35 dpi (bottom row) after gating on $CD3^-$ lymphocytes is depicted. (C) The frequency of NK cells recovered from the blood (C) and lung (D) 3, 10, and 35dpi following PBS (black bar), isotype (grey bar) or PK-136 (dark grey bar) treatment in x31 infected mice, with the total number of NK cells recovered from the lung quantified in (E). (F) Concentration of virus (pfu/mL) isolated from lung homogenates of PBS (black bar) or PK-136 (grey bar) treated mice 2 and 4 days following primary influenza infection with 10^3pfu x31 as determined by plaque assays on MDCK cells. Data are representative of 2 independent experiments using n = 3 mice/group. *$p<0.05$, **$p<0.01$, ***$p<0.001$, ****$p<0.0001$. Unpaired Student's t-test with Holm-Sidak multiple comparisons correction.

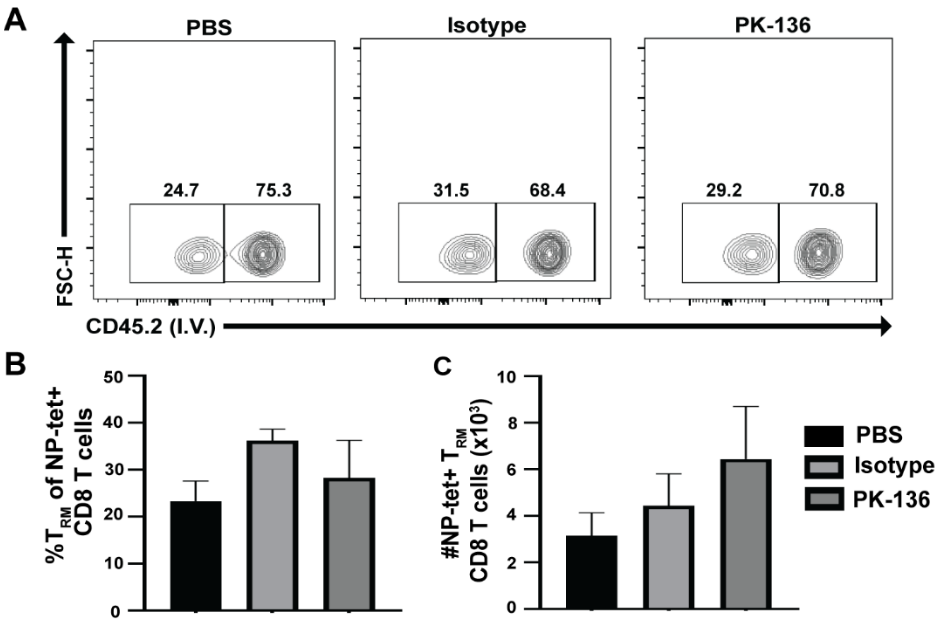

Supplemental Figure 3. NK depletion enhances the development of lung T_{RM} following influenza infection.

(A) C57BL/6 mice were infected with 10^3 PFU x31, administered 200ug anti-NK depleting antibody, isotype control IgG or PBS at -1, 1, and 3 dpi and rested for 35 days. T_{RM} were identified as cells which did not positively stain with an anti-CD45 administered i.v. 3 min prior to euthanasia, previously gated on single cells, lymphocytes, $CD8^+CD44^+NP\text{-}tet^+$ cells. The frequency (B), and number (C) of $NP\text{-}tet^+$ T_{RM} CD8 T cells ±SEM recovered from the lung of PBS (black), isotype (grey) or PK-136 (dark grey) treated animals is depicted. Graphs represent data from 1 independent experiments using n ≥ 4 mice/group/time point (total of 4 mice per group/time point). One-way ANOVA with multiple comparisons correction.
